# Clonal plasticity and diversity facilitates the adaptation of *Rhododendron aureum* Georgi to alpine environment

**DOI:** 10.1371/journal.pone.0197089

**Published:** 2018-05-10

**Authors:** Xiaolong Wang, Wei Zhao, Lin Li, Jian You, Biao Ni, Xia Chen

**Affiliations:** 1 National & Local United Engineering Laboratory for Chinese Herbal Medicine Breeding and Cultivation, School of Life Sciences, Jilin University, Changchun, Jilin province, People’s Republic of China; 2 Department of Biochemistry, Qiqihar Medical University, Qiqihar, Heilongjiang province, China; Aristotle University of Thessaloniki, GREECE

## Abstract

Four small oval populations and five large intensive populations of *Rhododendron aureum* growing at the alpine in Changbai Mountain (China) were studied in two types of habitat (in the tundra and in *Betula ermanii* forest). Identification and delimitation of genets were inferred from excavation in small populations and from amplified fragment length polymorphism (AFLP) markers by the standardized sampling design in large populations. Clonal architecture and clonal diversity were then estimated. For the four small populations, they were monoclonal, the spacer length (18.6 ± 5.6 in tundra, 29.7 ± 9.7 in *Betula ermanii* forest, P < 0.05) was shorter and branching intensity (136.7 ± 32.9 in tundra, 43.4 ± 12.3 in *Betula ermanii* forest, P < 0.05) was higher in the tundra than that in *Betula ermanii* forest. For the five large populations, they were composed of multiple genets with high level of clonal diversity (Simpson’s index D = 0.84, clonal richness R = 0.25, Fager's evenness E = 0.85); the spatial distribution of genets showed that the clonal growth strategy of *R*. *aureum* exhibits both guerilla and phalanx. Our results indicate that the clonal plasticity of *R*. *aureum* could enhance exploitation of resource heterogeneity and in turn greatly contribute to maintenance or improvement of fitness and the high clonal diversity of *R*. *aureum* increase the evolutionary rates to adapt the harsh alpine environment in Changbai Mountain.

## Introduction

Plant survival in alpine landscapes is constantly challenged by the harsh and often unpredictable environmental conditions[1]. Life cycles of alpine plants are threatened by the high uncertainty as to whether flowering and fruiting, germination and establishment can be successfully completed[2,3]. With increasing altitude and increasing latitude, perennial plants that reproduce clonally become more abundant[4]. In alpine and arctic areas, vegetative propagation plays an important role in reproduction, as more than 90% of species are clonal and can spread vegetatively by using organs such as rhizomes, stolons, or bulbils[3,5]. As clonal plants having the traits of resource storage, tight regulation of resource acquisition, and cycling and maintenance of dormant buds, which enable branching following death of apical meristems that help them to survive harsh climates and changing environmental conditions[6].

Clonal plants individuals can be recognized at two different organizational levels: genets and ramets. A genet is a group of genetically identical individuals, which have grown in a given location, all originating vegetatively, not sexually, from a single ancestor. The genet comprised of all tissues originating from one zygote, all genetically identical members of a clone, also called genetics individuals or clones, whereas a ramet is an individual or a potentially independent part of a clonal colony. A ramet is a potentially independent part of a genet. [7,8]. The spatial distribution and the degree of intermingling of clones will depend on the clonal growth strategy (clonal architecture) and two types of clonal growth strategy can be recognized: guerrilla and phalanx. In the guerilla growth form, longer internodes have more widely spaced ramets and will be more likely to intermingle with each other. In the phalanx growth form, plants produce shorter internodes, tend to form an advancing front of closely packed ramets, and produce clones that are juxtaposed. However, most species have a growth form that falls between these two extremes[9,10]. Individual organisms can alter their development, physiology and life history depending on environmental conditions[11]. Many clonal plants are able to respond to variation in environmental conditions by altering their clonal morphology in terms of spacer length and branching intensity[12,13]. This plasticity may be adaptive and may thus contribute to the spreading of the risk of genet extinction as well as to foraging for essential resources in heterogeneous environments[14,15].

A high degree of asexual reproduction is often associated with genetic monomorphism[10,16], however, many researchers have found that clonal populations can have high genetic diversity. Studies of the arctic-alpine *Carex bigelowii* Torr. ex Schwein and the alpine *Carex curvula* All. demonstrated high levels of clonal diversity[17–20]. Populations of *Rhododendron ferrugineum* L. intermediate to high levels of genetic diversity were detected based on AFLP molecular markers[21]. Such a high level of genotypic diversity has often been explained as a result of microsite heterogeneity that promotes the coexistence of clones through diversifying selection, as does frequency-dependent selection[17,22,23]. Moreover, seedling recruitment contribute to the clonal diversity[24,25]: (i) seedling establishment occurs heavily during a short period early in population development, with no further seedling establishment after the initial colonizing phase (Initial Seedling Recruitment: ISR), in which case, loss of genet diversity may occur with time and decrease the capacity of a population to respond to changing environmental conditions; (ii) seedling recruitment occurs repeatedly (RSR) during population development; and (iii) seedling recruitment occurs in a “window of opportunity” (RWO), episodically on a frequency scale of several decades or centuries depending on the perturbation.

Although excavation can directly reveal the extent and patterns of clonal plants, excavation is not effective over large areas due to the high cost, root fragmentation and root grafting. Moreover, root fragmentation removes the physical evidence of clonality, root grafting may connect non-clonal genets. Molecular methods are a major advance over phenological or root connectivity studies in the identification of clonal plants, and many studies have successfully used Amplified Fragment Length Polymorphisms AFLPs[26] to identify plant clones[21,23,27]. Though the polymorphic information content of AFLPs is generally lesser than that of microsatellites, the microsatellites is cumbersome and laborious requiring screening of thousands of genomic clones through hybridization using short radiolabeled microsatellite probes[28]. AFLP is a fast, efficient and superior technique compared to isoezyme and RAPD. Many markers can be scored for each sample in a single run, using an automatic genetic analyser[29].

*Rhododendron aureum* Georgi (syn. *Rh*. *Chrysanthum* Pall.), the target species in this study, is a perennial evergreen dwarf shrub with well-branched trailing stems inhabiting alpine regions of Korea, China, Japan, and the Kamchatka peninsula. This plant is approximately 1 m high, blooms from June to July, and the flowers have a pale yellow color. It has been shown that occupies the snowmelt gradient and especially to dominate in early exposed places[30]. These attributes suggest that the species *R*. *aureum* exhibits morphological and behavioral adaptations to the alpine environment[31]. In China, it is native to the alpine tundra and under the *Betula ermanii* forest of Changbai Mountain, altitude ranging from 1,000 to 2,506 m a.s.l.[32]. The record of recent years shows that it distributed in altitude of 2600 m in the Changbai Mountains[33]. The *R*. *aureum* is one of the constructive and dominant species on the alpine ecosystem in Changbai Mountain, and it plays an important role in maintaining the ecological balance by preventing and controlling soil erosion. For *R*. *aureum*, few seedling establishment has been observed in Changbai Mountain [34], and we have found some small oval populations that may be monoclonal populations in the wild. That means *R*. *aureum* may be similar to *R*. *ferrugineum* which is clonal reproduction in alpine[21]. However, there is little known about the clonal reproduction, clonal growth strategy and clonal diversity of *R*. *aureum*.

The aims of this study were (i) to identify the clonal reproduction in candidate monoclonal populations; (ii) to determine the clonal growth strategy; (iii) to define the clonal diversity and spatial distribution of clones in candidate multiclonal populations and (iv) to discuss the effect of clonality on adaptation to alpine environment. These aims were addressed in four candidate monoclonal populations and five candidate multiclonal populations in Changbai Mountain using field investigation and AFLP markers.

## Materials and methods

### Study sites

This study was conducted on the northern and western slope of Changbai Mountain, which is generally acknowledged as the highest mountain in northeast China and eastern Eurasia. The region is a typical mountain climate. The climate conditions are also distinct in different altitudes. The average annual temperature is generally 3°C–7°C, and annual precipitation is over 600 mm. With relatively high altitude above sea level, the annual precipitation is over 1400 mm[35]. The alpine region of Changbai Mountain is the southern boundary of alpine tundra in East Eurasia. Varied topography, weather, soil and other natural conditions result in rich biodiversity and vertical zonal distribution of vegetation on Changbai Mountain, where harbors over 2,277 species of plants and a notable richness of endemic species. The sampling sites were distributed in alpine tundra and the *Betula ermanii* forest with altitude range from 1800m to 2600m.

### Sampling strategy

Four candidate monoclonal populations (DX1-DX4) which the shape of the population on the ground is oval (hereafter this text will be abbreviated as oval populations) with diameter range from 1.3m to 1.9m of *R*. *aureum* were investigated by excavation in the field during the summer of 2015. DX1 and DX2 were located in the *Betula ermanii* forest; DX3 and DX4 were located in the alpine tundra. The spacer length, branching intensity and branching angle was measured. We have re-transplant the *R*. *aureum* back to the original place immediately to reduce the damage to these populations. The leaves from ten ramets in each candidate monoclonal population were sampled and dried directly in silica gel. The candidate multiclonal populations for investigating clonal propagation and clonal diversity was carried out in large and dense populations on flat areas with no intervening larger patches of other species or bare ground. Four rectangular sampling plots and one linear arrangement plot were selected on Changbai Mountain to assess clonal propagation and clonal diversity of the population. CN1, CN2, CN3 was located in the north slope of Changbai Mountain and CW1 and CW2 located in the western slope. Populations CN3 and CW1 were in the *Betula ermanii* forest, the rest populations were on the tundra. The geographic coordinates for all populations were shown in **Table 1**. As population CN1 was roughly distributed in a linear arrangement along the terrain, we obtained 36 samples along the terrain. Within the other plots, 35 to 44 leaves (one per branch) of *R*. *aureum* were collected at each point of intersection of a 1 m × 1 m grid and immediately preserved in silica gel to prevent DNA degradation.

**Table 1:** The geographic coordinates for all populations.

Population	GPS Coordinates		Altitude
	longitude	latitude	(m)
DX1	42°03′20.24″	128°04′12.75″	1943
DX2	42°03′19.70″	128°04′15.42″	1948
DX3	42°02′41.53″	128°04′31.81″	2206
DX4	42°03′07.98″	128°04′29.30″	2059
CN1	42°01′46.71″	128°03′59.97″	2600
CN2	42°04′04.03″	128°03′24.96″	2300
CN3	42°03′34.39″	128°03′46.88″	1800
CW1	41°59′24.76″	128°00′10.42″	2042
CW2	41°59′28.83″	128°01′01.24″	2223

### DNA extraction and AFLP procedure

Total DNA was extracted from silica-dried leaves using a Plant Genomic DNA Kit (Bioteke Beijing Co. Ltd., Beijing, China). DNA samples were diluted to 10 ng/μl and then stored at -20°C until further analysis.

The amplified fragment length polymorphism (AFLP) method developed by Vos et al[26] was performed with the following modifications: restriction digestion and ligation were performed simultaneously in a 10 μl solution containing 50 ng of genomic DNA, 1 U of *EcoRI* (Fermentas, Shenzhen, China), 1 U of *MseI* (New England Biolabs (Beijing) LTD), 1μl of 10 × restriction–ligation buffer, 1 U of *T4* DNA ligase(Fermentas, Shenzhen, China), 0.2 mM of ATP, 1.0 μM of *MseI* adapter and 0.1 μM of *EcoRI* adapter and double-distilled water. The mixture was incubated at 37°C for 8 h, 16°C for 4 h, inactivated at 65°C for 10 min, and stored at 4°C. Pre-amplification was performed in a 25μl solution containing 2.5μl of diluted restriction–ligation product, 0.2 μM of dNTPs, 0.3 μM of each primary amplification primer, 2.5 μl of 10× PCR buffer and 0.5 U of Taq polymerase (Transgen Biotech Beijing Co. Ltd., China). The pre-amplification conditions consisted of pre-denaturation for 5 min at 94°C, followed by 30 cycles of denaturation for 30 s at 94°C, annealing for 60 s at 56°C, and elongation for 80 s at 72°C, with a final extension for 10 min at 72°C and storage at -20°C. Products were diluted 1 to 40 (v/v) with ddH_2_O. The optimized selective amplification PCR reaction system (25 μl) for *R*. *aureum* 2 μl diluted pre-amplification product, 0.2 mM dNTPs, 2 μM *EcoR I* and *MseI* selective primer, 2.5μl of 10× buffer and 0.5 U of Taq polymerase and double-distilled water. We pre-screened 64 selective primer pairs and chose ten pairs that were reliable for this study. The detail sequences of primers were in **S1 Table**. The selective PCR reaction had two cycle sets: 13 cycles of 30 s at 94°C, 30 s at 65°C (annealing temperature was lowered 0.7°C at each cycle) and 60 s at 72°C, followed by 18 cycles of 30 s at 94°C, 30 s at 56°C and 80 s at 72°C. After selective amplification, the products were mixed 1:1 with a loading buffer (98% deionized formamide, 10 mM EDTA, 0.1% bromophenol blue, and 0.1% xylene cyanol), denatured at 95°C for 5 min, and separated on 6% denaturing polyacrylamide gels (6% polyacrylamide and 7 M urea) in 1× TBE buffer at 70 W for 4.5 h. Gels were stained according to the silver staining method[36]. To test the repeatability of AFLP results, two individuals from each population were completely replicated starting from the restriction/ligation reaction of AFLP.

### Data analysis

#### Clonal growth strategy

Tukey’s LSD post hoc test was used to test the significant differences of spacer length, branching intensity and branching angle between different populations. Statistical analyses were performed using 19.0 SPSS for Windows (Chicago, SPSS Inc.).

#### Clones detection

AFLP-amplified DNA fragments (bands) were scored as present (1) or absent (0), and a data matrix of the AFLP banding patterns of all populations was assembled for further analysis. The program POPGENE v1.31 [37] was used to estimate the following genetic diversity parameters: Shannon’s information index (*I*) [38], percentage of polymorphic loci (*PPL*), genetic distance and genetic identity. The clonal structure identification followed the method of Mejías and Vonlanthen [39,40]. Based on the average genetic distance within the monoclonal populations, we have set the maximum genetic distance threshold for sample assignment to the same clone. Samples were assigned to the clone of the central individual by means of a hierarchical cluster analysis based on genetic distance[41] using NTsyspc v2.02 [42].

#### Clonal diversity

To estimate clonal diversity, four frequently used measures were calculated following Ellstrand and Roose[43]:

(1) The number of genets (G)

(2) Simpson diversity index (*D*)[44]:

$$D=1-\frac{\sum \left(N_{i}(N_{i}-1)\right)}{N(N-1)}$$ (1)

where *Ni* is the number of individual shoots with AFLP phenotype *i* and N is the total number of samples. This measure describes the clonal heterogeneity in a population; it ranges from 0 to 1. When *D* = 0, a population is composed of a single clone; when *D* = 1, a population has no clonal growth.

(3) Fager's evenness (*E*)[45]:

$$E=\frac{D-D_{min}}{D_{max}-D_{min}}$$ (2)

where $D_{min}=\frac{\left(G-1)(2N-G\right)}{N(N-1)},\;D_{max}=\frac{(G-1)N}{G(N-1)}$; it ranges from 0 to 1. When *E* = 0, indicating that the entire population has only one clone or the most ramets belong to one clone while the other clone only contain one ramets; when *E* = 1, there are same ramets of different clones within the population.

(4) The clonal richness (*R*)[46]:

$$R=\frac{G-1}{N-1}$$ (3)

such that the smallest possible value, a monoclonal stand is 0, independently of the sample size. Maximum clonal richness is 1, when all different samples correspond to different genotypes.

## Result

**Fig 1:**
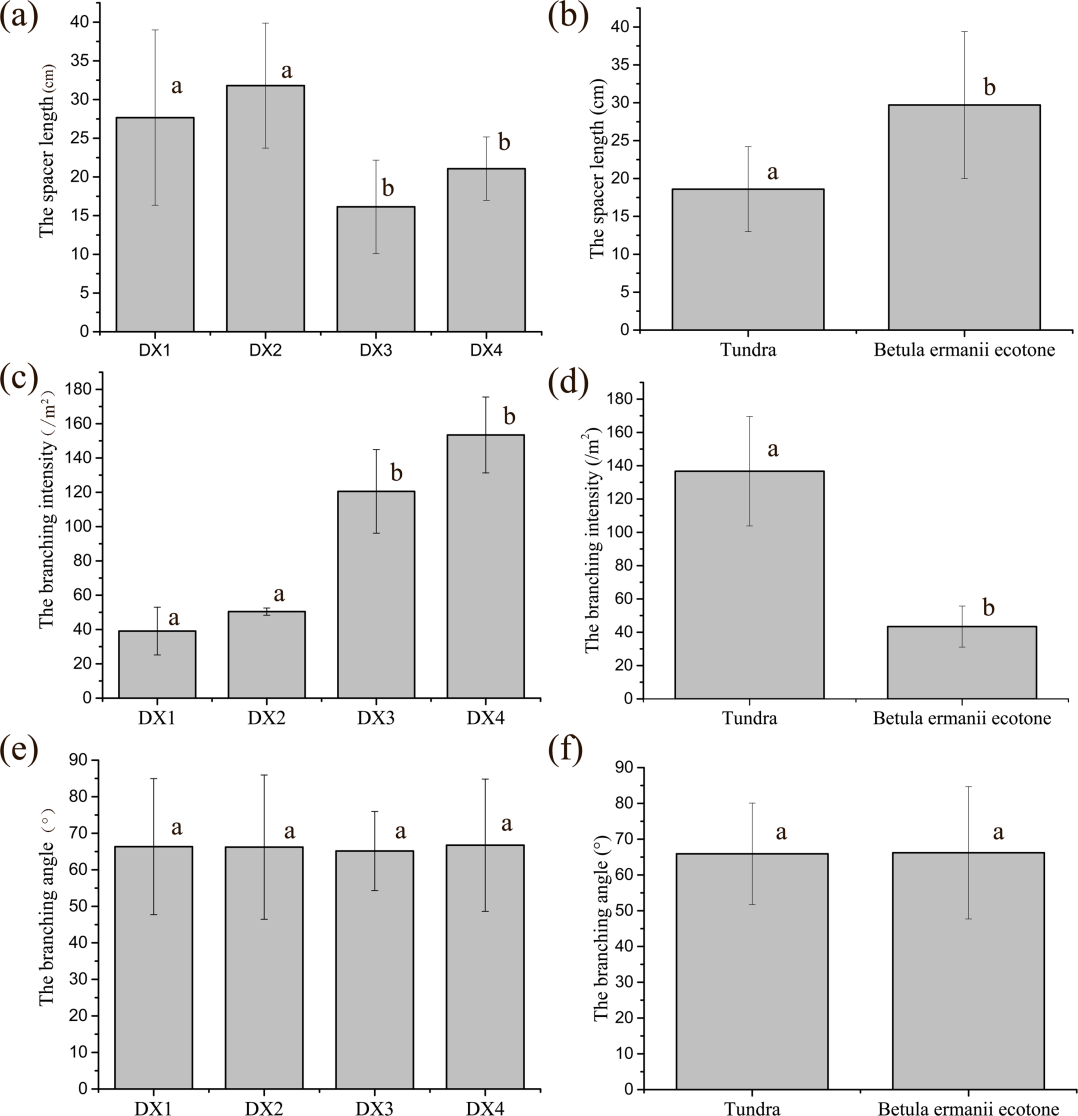
The clonal growth form of monoclonal populations in the tundra and *B*. *ermanii* forest. There were significant differences among the populations with the different letters (*P* < 0.05), and there were no significant differences among the populations with the same letter. Bars are standard errors.

The four small oval populations (DX1-DX4) are indeed monoclone revealed by excavation. The average spacer length, branching intensity and branching angle of the four monoclonal populations is 24.17 ± 9.77 cm, 90.86 ± 50.78 branches/m^2^, 66.10 ± 16.75°respectively. There are some difference between the different populations on the spacer length and branching intensity, whereas the branching angle was similar among the four populations, showed in **Fig 1**. In DX1 and DX2, the spacer length was longer, but the branching intensity was lower than that in DX3 and DX4 significantly (*P* < 0.05, **Fig 1A and 1C**). As DX1 and DX2 were located in the *B*. *ermanii* forest, DX3 and DX4 were located in the alpine tundra; thus monoclonal populations located in the tundra showed the shorter spacer length and higher branching intensity than that the monoclonal populations located in *B*. *ermanii* forest and there was no difference of branching angle between the two habitat types (**Fig 1B, 1D and 1F**).

**Fig 2:**
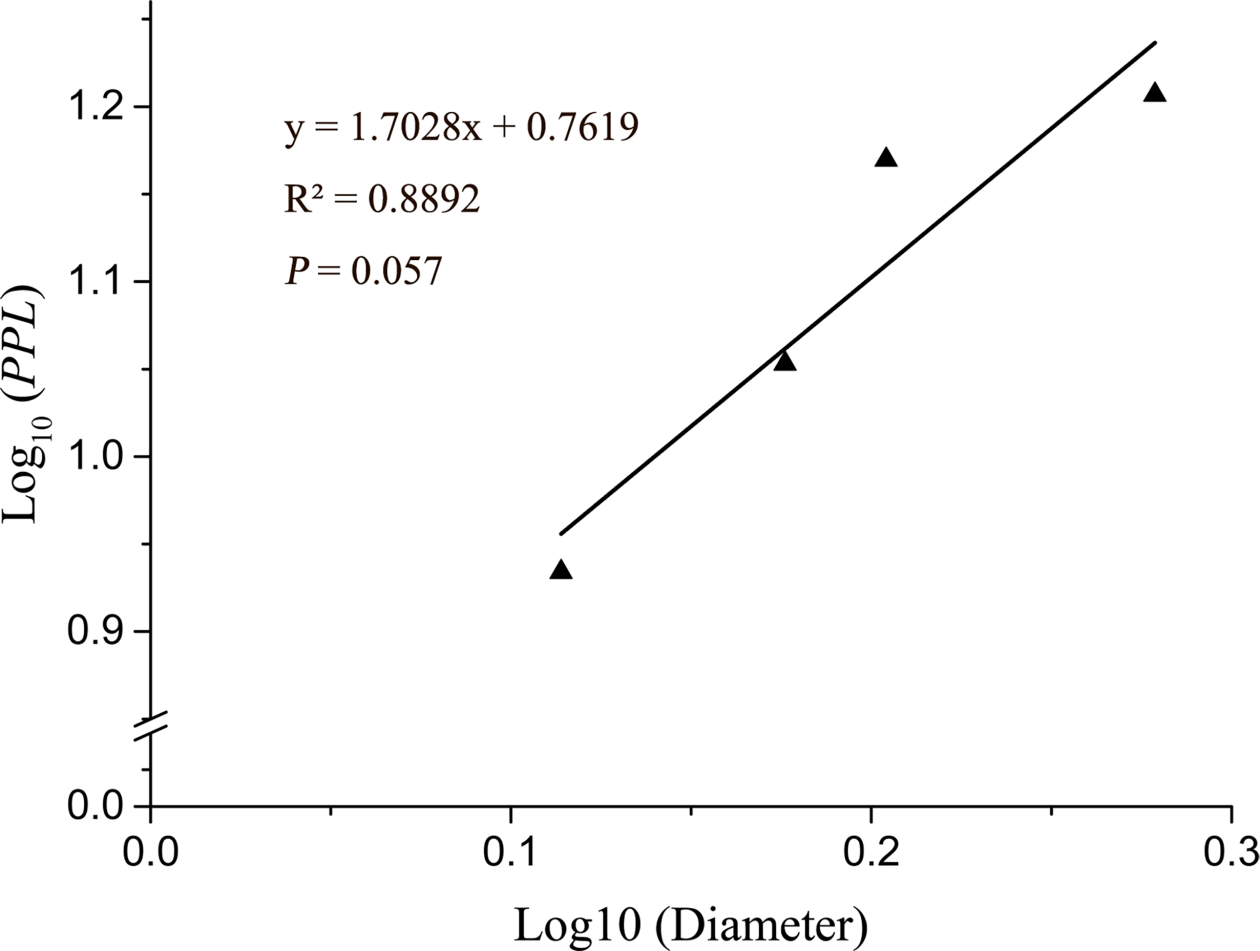
Relationship between population diameter and genetic variation in monoclonal populations.

For the 40 samples from four monoclonal populations, AFLP analysis was used to detect the threshold value which determines ramets belonging to a same clonal lineage. The number of unambiguously scorable fragments generated by ten AFLP primer combinations range from 291 to 292 in 40 individuals from 4 monoclonal populations, molecular size of the fragments ranging from 100 to 1500bp (**Table 2**). The minimum genetic identity ranged from 0.952 to 0.977 with average of 0.962 and the maximum genetic distance ranged from 0.023 to 0.048 with average of 0.038. Here we set the genetic identity threshold and genetic distance threshold to 0.962 and 0.038 respectively, which means that samples which genetic identity more than 0.962 and genetic distance less than 0.038 were defined as the same clones in multiclonal populations. We have found that the PPL and diameter of monoclonal populations were positively correlated **(Fig 2)**.

**Table 2:** The genetic diversity index and population diameter of *R*. *auruem* monoclonal populations.

population	DX1	DX2	DX3	DX4	mean
N	10	10	10	10	10
PPL	11.3	16.1	14.78	8.59	12.69
The number of bands	292	292	291	291	291.5
maximum genetic distance	0.038	0.048	0.044	0.023	0.038
minimum genetic identity	0.962	0.952	0.956	0.977	0.962
diameter/m	1.5	1.9	1.6	1.3	1.58

Note: N, number of samples; PPL, polymorphic loci percentage.

**Fig 3:**
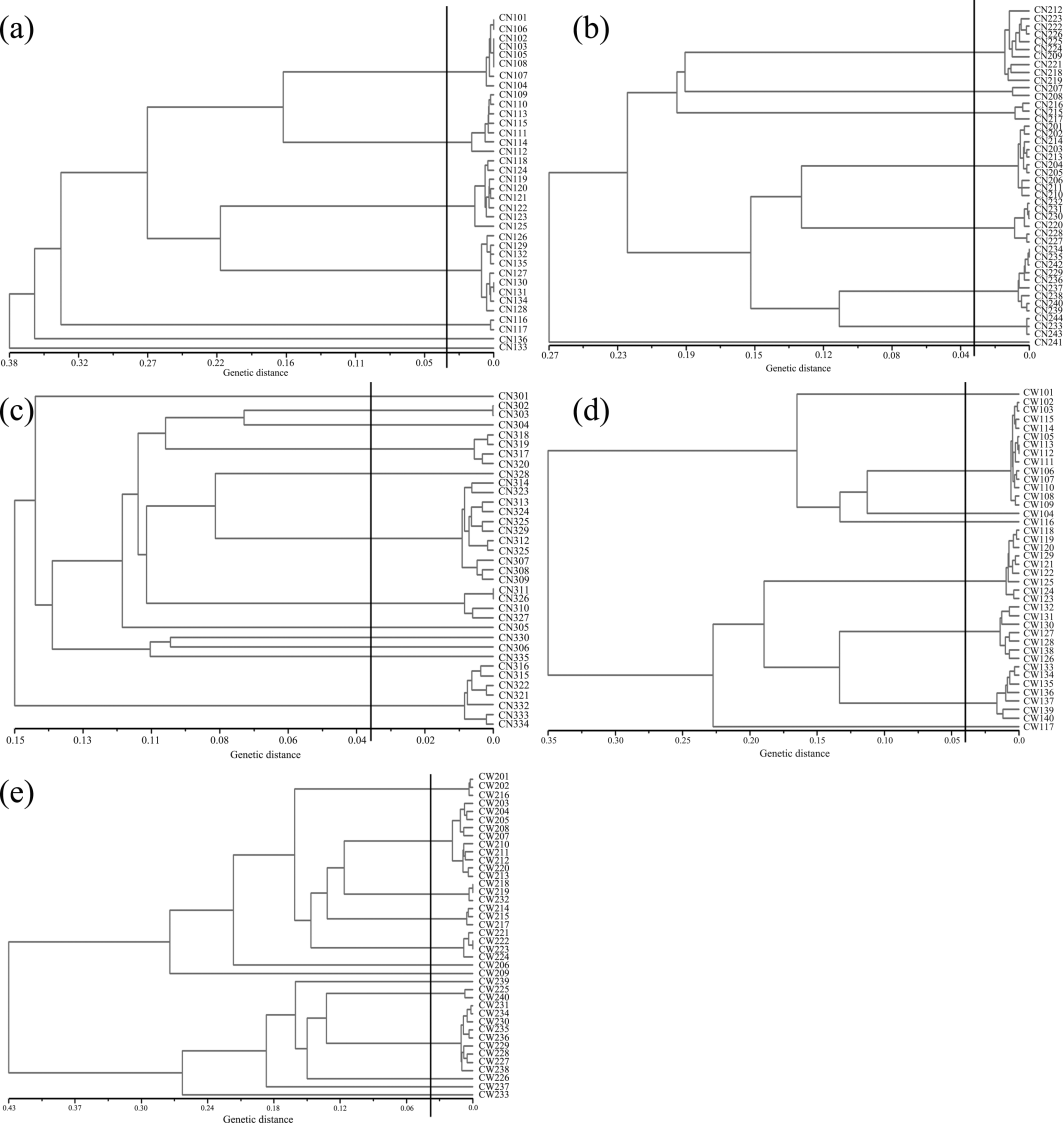
Identify clones by hierarchical cluster analysis based on the AFLP markers. The vertical line shows the genetic distance threshold for identifying the same clone, a-e represented the populations CN1, CN2, CN3, CW1, CW2 respectively.

**Fig 4:**
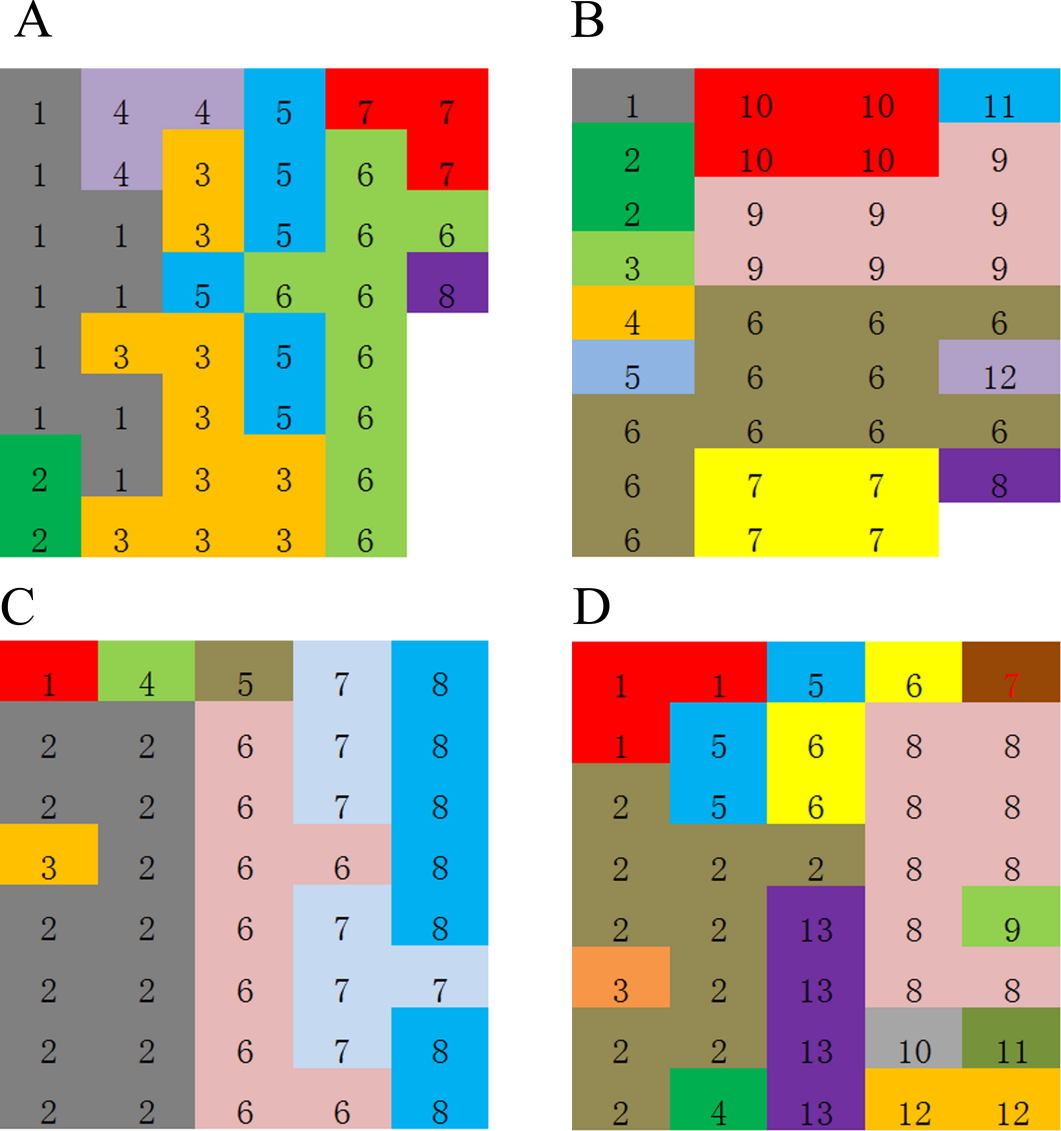
Map showing the spatial distribution of *R*. *aureum* genets in the muticlonal populations. Sampling distance is 1.0 m and genets are shown in different colours. A-D represented the populations CN2, CN3, CW1 and CW2 respectively.

Based on the genetic identity and genetic distance threshold, the clone identification results are shown in **Fig 3**. We detected 7–13 clones out of the five multiclonal populations, with a mean of 9.8 genets **(Table 3).** The average size of each genotype ranged from 2.92 to 5.14, with a mean of 4.21. *R*. *aureum* exhibits a high level of clonality, but is also genetically diverse. Clonal diversity was rather high in all five multiclonal populations (*D* = 0.80–0.88), with high clonal evenness (*E* = 0.79–0.89). The clonal diversity is similar between the populations growing in the tundra (CN1, CN2, CW2) and the populations growing in *B*. *ermanii* forest (CN3, CW1). Maps showing the spatial distribution of the samples assigned to genets in CN2, CN3, CW1 and CW2 populations are shown in **Fig 4**. As population CN1 was roughly distributed in a linear arrangement from CN101 to CN136 according to the terrain, we can see the spatial distribution in **Fig 3A**. The samples belonging to each genet were usually spatially grouped and in many cases, samples belonging to the same genet were separated by other genets. Indicating that the clonal growth strategy of *R*. *aureum* exhibits both guerilla and phalanx. Thus the species exhibits a transitional clonal growth form.

**Table 3:** Descriptive statistics of clonal diversity parameters in the investigated populations.

population	Altitude/m	N	G	D	R	E
CN1	2600	36	7	0.82	0.17	0.89
CN2	2300	44	9	0.85	0.18	0.89
CN3	1800	35	12	0.86	0.32	0.79
CW1	2042	40	8	0.8	0.18	0.83
CW2	2223	40	13	0.88	0.31	0.84
total/mean	—	195	49/9.8	0.84	0.25	0.85

Note: N, number of samples; *G*, number of genets; *D*, Simpson diversity index; *R*, clonal richness; *E*, Fager's evenness.

## Discussion

We predominantly observed clonal propagation on Changbai Mountain of *R*. *aureum*. In alpine ecosystems, with increasing altitudes, plant life is challenged by low temperatures, a shorter vegetation period, more snow, and harsher conditions. Life cycles of alpine plants are threatened by a high degree of uncertainty as to whether flowering, fruiting, germination, and establishment can be successfully completed [47,48]. Perennial plants that reproduce clonally increase in abundance with altitude [21,49]. Clonal growth, the ability for vegetative reproduction to occur by rhizomes or aboveground stolons, is one of the most remarkable adaptations to alpine conditions [1]. *R*. *aureum* exhibits a high level of clonality and exhibits transitional clonal growth strategy on Changbai Mountain. The study regions have a temperate continental climate with long cold winters, short warm summers, and extremely low mean annual temperatures that restricted the life cycle of *R*. *aureum*. Moreover, *R*. *aureum* blooms for only about a week, with flowering times ranging from June at low altitudes to August at high altitudes. *R*. *aureum* exhibits low selfing ability, and it is pollinated mostly by insects[50]; however, the early flowering populations have low fruit set, as pollinator activity is reduced under the low temperatures in mid-June. Later-flowering populations also failed to set fruits because of the onset of autumn frost and snow before fruit maturation [31]. As a result of the increasing risk of not completing the life cycle in time, the relative importance of sexual reproduction versus clonal reproduction might change.

Clonal propagation is essential for *R*. *aureum* to survive since clonal plants can adapt to heterogeneous environments by risk spreading among genets [51,52], clonal integration [53–55], foraging behavior [56–58], and division of labor [59]. The morphology of clonal plants, especially the morphological traits that determine the placement of cloned ramets in horizontal spaces, can react to the environment (such as light intensity and quality) conditions[60,61]. The spacer length and branching intensity of *R*. *aureum* showed significant differences in the tundra and in *B*. *ermanii* forest. In the tundra of Changbai Mountain, there are abundant light resources as no high tree cover, the *R*. *aureum* populations showed high degree of intensity; whereas in the *B*. *ermanii* forest, lighting resources are limited as the cover of *B*. *ermanii*, in order to obtain more light resources, the *R*. *aureum* populations was sparse. Clonal plasticity could enhance exploitation of resource heterogeneity by clonal plants, and in turn greatly contribute to maintenance or improvement of fitness[57]. Shrubs can grow through clonal means to form large patches across landscapes. The transition clonal growth form of *R*. *aureum* showed highly plastic changes in morphology of individual ramets that enable effective exploitation of local concentrations of essential resources once they have been located [62].

Populations of clonal plants consisting of few genets are subject to similar genetic processes that affect population, such as genetic drift and inbreeding which may lead to loss of genetic variability and may cause inbreeding depression [63,64]. Though we predominantly observed clonal propagation (**Table 3**) on Changbai Mountain, within-population clonal diversity was rather higher than the average for clonal species (*D* = 0.62, *E* = 0.68) as revealed by Ellstrand [43]. In many studies also showed that populations of clonal plants exhibit considerable levels of genetic diversity [17,65–67]. It has been suggested that this is also true for long-lived clonal plants from alpine habitats [19,68,69]. Most clonal plants maintain sexual reproduction[70], and their populations can therefore be as diverse as those of nonclonal plant species [1,71]. Several mechanisms for maintenance of diversity within clonal populations have been proposed including mutation, the generation of new genotypes by sexual members of the population or by sexual progenitor species, and differential selection in temporally or spatially heterogeneous environments[43,72]. In *R*. *aureum*, we have found genetic diversity even in populations consisting of a single clone, with variation that was proportional to the population diameter (**Fig 2**). Somatic mutations and diffuse centromeres can contribute to standing genetic variation in populations. Genetic diversity is key for a species to survive and adapt to changing environments [73–76] and the high genetic diversity of *R*. *aureum* increase the evolutionary rates to adapt the harsh alpine environment in Changbai Mountain.

## Conclusions

In our study we have found the clonal reproduction of *R*. *aureum* was widespread in Changbai Mountain reviewed by excavation and AFLP markers. The ramets of monoclonal populations arranged more intensive in tundra than that in the *B*. *ermanii* forest. The clonal growth strategy of *R*. *aureum* exhibits both guerilla and phalanx. *R*. *aureum* showed high level clonal plasticity in different habitats. The plasticity of *R*. *aureum* allows them to change their growth form so that they could adapt to alpine heterogeneous habitat. *R*. *aureum* exhibit a high level of genetic variation within populations. With the genetically more diverse, *R*. *aureum* have ability to buffer the effects of poor environmental conditions in alpine and increase the evolutionary rates to adapt the harsh alpine environment in Changbai Mountain.

## Supporting information

S1 Table The primers used for AFLP analysis. (DOCX)
