# Supplementary material for: Clonal plasticity and diversity facilitates the adaptation of Rhododendron aureum Georgi to alpine environment
Source: PLoS One. 2018 May 10;13(5):e0197089. doi: 10.1371/journal.pone.0197089 (PMC5944948; doi:10.1371/journal.pone.0197089)
Supplement: S1 Table — (DOCX) [file pone.0197089.s001.docx]

**Local adaptation to alpine environment in naturally fragmented populations of *Rhododendron aureum* Georgi in Changbai Mountainan, China**

**Xiaolong Wang^1,2^, Jianwei Lv^3^, Wei Zhao^1,2^, Lin Li^1,2^, Xia Chen^1,2,^***

1. National & Loal United Engineering Laboratory for Chinese Herbal Medicine Breeding and Cultivation, School of Life Sciences, Jilin University, Changchun, Jilin province,130012, People’s Republic of China.

2. School of Life Sciences, Jilin University, Changchun, Jilin province, 130012, China.

3. Huhhot Vocational College, Huhhot, Inner Mongolia, 010051, China.

* For correspondence. E-mail address: [chenxiajlu@163.com](mailto:chenxiajlu@163.com)

Table S1 The primers used for AFLP analysis

| primer | forward primer | reverse primer |
| --- | --- | --- |
| Pre- amplification | GACTGCGTACCAATTCA | GATGAGTCCTGAGTAAC |
| AFLP-1 | GACTGCGTACCAATTCACC | GATGAGTCCTGAGTAACAA |
| AFLP-2 | GACTGCGTACCAATTCACC | GATGAGTCCTGAGTAACTA |
| AFLP-3 | GACTGCGTACCAATTCAGG | GATGAGTCCTGAGTAACAT |
| AFLP-4 | GACTGCGTACCAATTCAGG | GATGAGTCCTGAGTAACAG |
| AFLP-5 | GACTGCGTACCAATTCACA | GATGAGTCCTGAGTAACTA |
| AFLP-6 | GACTGCGTACCAATTCACA | GATGAGTCCTGAGTAACTT |
| AFLP-7 | GACTGCGTACCAATTCACT | GATGAGTCCTGAGTAACAA |
| AFLP-8 | GACTGCGTACCAATTCACT | GATGAGTCCTGAGTAACAT |
| AFLP-9 | GACTGCGTACCAATTCACT | GATGAGTCCTGAGTAACAC |
| AFLP-10 | GACTGCGTACCAATTCACT | GATGAGTCCTGAGTAACTA |
